# A Study of the CO Sensing Responses of Cu-, Pt- and Pd-Activated SnO_2_ Sensors: Effect of Precipitation Agents, Dopants and Doping Methods

**DOI:** 10.3390/s17051011

**Published:** 2017-05-03

**Authors:** Venkata Krishna Karthik Tangirala, Heberto Gómez-Pozos, Ventura Rodríguez-Lugo, María De La Luz Olvera

**Affiliations:** 1 Departamento de Ingeniería Eléctrica-SEES, Centro de Investigación y de Estudios Avanzados del Instituto Politécnico Nacional, CINVESTAV-IPN, Apartado postal 14740, México D. F. 07360, Mexico; molvera@cinvestav.mx; 2 Área Académica de Computación y Electrónica, ICBI, Universidad Autónoma del Estado de Hidalgo, Hidalgo 56092, Mexico; gpozos@uaeh.edu.mx; 3 Área Académica de Ciencias de la Tierra y Materiales, Instituto de Ciencias Básicas e Ingeniería, Universidad Autónoma del Estado de Hidalgo, Carretera Pachuca-Tulancingo Km. 4.5, Hidalgo 42184, Mexico; ventura.rl65@gmail.com

**Keywords:** tin oxide pellets, doping, HRTEM analysis, CO, sensing response

## Abstract

In this work, we report the synthesis of Cu, Pt and Pd doped SnO_2_ powders and a comparative study of their CO gas sensing performance. Dopants were incorporated into SnO_2_ nanostructures using chemical and impregnation methods by using urea and ammonia as precipitation agents. The synthesized samples were characterized using X-ray diffraction (XRD), Raman spectroscopy, scanning electron microscopy (SEM) and high resolution transmission electron microscopy (HR-TEM). The presence of dopants within the SnO_2_ nanostructures was evidenced from the HR-TEM results. Powders doped utilizing chemical methods with urea as precipitation agent presented higher sensing responses compared to the other forms, which is due to the formation of uniform and homogeneous particles resulting from the temperature-assisted synthesis. The particle sizes of doped SnO_2_ nanostructures were in the range of 40–100 nm. An enhanced sensing response around 1783 was achieved with Cu-doped SnO_2_ when compared with two other dopants i.e., Pt (1200) and Pd:SnO_2_ (502). The high sensing response of Cu:SnO_2_ is due to formation of CuO and its excellent association and dissociation with adsorbed atmospheric oxygen in the presence of CO at the sensor operation temperature, which results in high conductance. Cu:SnO_2_ may thus be an alternative and cost effective sensor for industrial applications.

## 1. Introduction

Gas leak detection is a constructive testing methid for dangerous combustible gases [1]. Carbon monoxide (CO) is a toxic hazardous industrial gas produced from the incomplete burning of all carbon-based fuels. Different kinds of materials including metal oxide semiconductors (MOSs) are employed for detecting CO [2,3,4,5,6,7,8]. As Wagner discovered [9], atoms and molecules adsorbed on the MOS surface influence its conductivity. Among the available MOS materials, SnO_2_ is usually considered to be one of the best candidates for developing gas sensors due to its relatively low cost of production and adequate oxygen vacancies [10,11].

Commercially available SnO_2_ gas sensors are mainly in the form of thick or thin films or pellets [12,13]. Despite having disadvantages like high power consumption, low temperature homogeneity and low electrode efficiency in collecting the free charge carriers, pellets are more viable for gas sensors due to their high porosity, abundant material, no substrate effects and they can serve as reference values for comparing thin or thick film sensors. Dopants in MOS materials improve the surface reactivity with atmospheric oxygen and subsequently the sensing response by residing on the surface in the form of metal clusters or by modifying the crystalline structure [14,15]. For gas sensing applications, the major catalytically active doping additives are noble metals (Pd, Pt, etc.), and transition metals (Cu, Fe, etc.) [16,17,18]. In general transition metals serve as “accelerators” of various processes [19] and noble metals serve as “catalysts” and also decrease the sensor operation temperature [20]. Platinum (Pt) and palladium (Pd) are the most used dopants due to their chemical inertness and higher work function (~5.8 and ~5.4 eV, respectively) than the band gap (3.6 eV) of SnO_2_ [21,22,23,24,25]. On the other hand, copper (Cu) is the most used transition metal for doping due its comparable ionic radius as tin. The radii of Sn^4+^ and Cu^2+^ are around 0.71 and 0.72 Å, respectively [26]. The association of Cu with atmospheric oxygen and its dissociation with CO at elevated temperatures also improves the surface conductivity of Cu-doped SnO_2_ pellets [27].

Different methods like flame spray [28], sol-gel [29], microwave irradiation [30], and wet chemical synthesis [31] are reported for the preparation of SnO_2_ powders. Among others, wet chemical synthesis employing urea as precipitant agent requires a moderate temperature (80–100 °C) which provides coarse powders with adequate characteristics to be used in gas sensing applications [32]. Another key factor that affects the sensing response is the dopant concentration. Many previous works about doping concentration effects reason that the sensing response increases with the increase in the doping concentration due to the corresponding increase in catalytical activity [26,27]. In this work, a study of dopant concentration effect is obviated due to the consideration of adequate variables like precipitation agent—urea and ammonia, dopant type—Cu, Pt and Pd and doping method—chemical doping and impregnation methods. In our future work these sensors will be tested for different dopant concentrations for different gas atmospheres to obtain selectivity and stability.

In this work, SnO_2_ pellets doped with 1 wt % Cu, Pt and Pd, were synthesized by wet chemical synthesis using urea and ammonia as a precipitation agents. Ammonia was employed to observe the effect of precipitation agent in the particle size. Additionally, two methods were employed for the incorporation of the dopants Cu, Pt and Pd, namely, chemical doping and the impregnation method. Cu- doped SnO_2_ pellets show excellent gas response to CO as comparison Pt- and Pd-doped SnO_2_ pellets using both doping methods. Simultaneously, the structural, morphological characterizations and their corresponding calculations provide a clear and deep perception of the effects of the dopant on the sensing properties of SnO_2_. Our study thus represents a viable way for understanding the effect of dopants on the SnO_2_ gas sensors.

## 2. Materials and Methods

### 2.1. Preparation of Former Pure SnO_2_ Powders Using Urea and Ammonia

Feedstock solution was prepared by mixing 0.4 M aqueous tin chloride pentahydrate (SnCl_4_·5H_2_O; J. T. Baker, Mexico City, Mexico and urea (CH_4_N_2_O; Sigma Aldrich, Mexico City, Mexico in 1:2 ratio. Then the mixed solution was vigorously stirred and heated until the solution temperature reaches around 93 ± 5 °C. Unlike the former case (urea), for ammonia (NH_4_OH; Sigma Aldrich) as precipitation agent it was added dropwise to 0.4 M aqueous SnCl_4_·5H_2_O until the pH of the solution reached 12. The resultant precipitates in both cases were centrifuged at 400 rpm for 1 h using a ROTINA-420R centrifuge (Hettich, Toluca, Mexico) and the obtained pastes were dried at 100 °C for 24 h to eliminate any remaining solvent. Finally, the dried powders were calcined in a furnace at 800 °C for 2 h to obtain pure SnO_2_ powders. All the synthesis conditions were studied, optimized and reported in our previous works [33,34].

### 2.2. Preparation of Chemical Doped SnO_2_ Powders

One wt % aqueous CuCl_2_ (Sigma Aldrich) was added to a previously prepared stock solution (0.4 M aqueous SnCl_4_·5H_2_O and CH_4_N_2_O in 1:2 ratio). Then a procedure similar to the one explained in Section 2.1 was followed to obtain Cu-doped SnO_2_ powders (Cu:SnO_2_) utilizing urea and ammonia as precipitation agents. Similarly, Pt:SnO_2_ and Pd:SnO_2_ powders were prepared utilizing PtCl_2_ and PdCl_2_, respectively.

### 2.3. Preparation of Impregnated SnO_2_ Powders

Primarily, 1 g each of the previously obtained (refer to Section 2.1) pure SnO_2_ powders utilizing the precipitation agents urea and ammonia were considered. Subsequently, these powders were separately impregnated with 1 wt % aqueous solutions of dopant chlorides, CuCl_2_, PtCl_2_ and PdCl_2_ (Sigma Aldrich). All the impregnated powders were annealed at 350 °C for 2 h in air to remove the residual species. A summary of all the samples obtained by the different doping methods and using the different precipitation agents is listed in Table 1. Pellets were manufactured from all the obtained Cu-, Pt- and Pd- doped SnO_2_ powders by using a manual pressing machine. The optimal pressing conditions, determined after several trials, were 16 tons applied for 90 min.

### 2.4. Characterization

X-ray diffraction analysis using a PANalytical diffractometer (Panalytical, Mexico City, Mexico) with CuKα at 20 mA and 40 kV was carried out to identify the phase compounds, crystallite size, lattice parameters and the crystalline structure of the doped SnO_2_ powders. Raman scattering spectroscopy of pure and doped SnO_2_ powders were analyzed using 532 nm laser beams and detecting scattering signals by a thermoelectrically cooled charge coupled detector.

Scanning electron microscopy (SEM), using an AURIGA instrument (Zesis, Mexico City, Mexico), was employed to examine the morphological characteristics and the particle size of the calcined agglomerates. Additionally, SEM was also employed to analyze the surface morphological characteristics of all synthesized powders. A high resolution transmission electron microscope (HRTEM, JEM-ARF 200F, JEOL, Mexico City, Mexico) was employed to identify the dopants; additionally crystallite size, crystal planes and the lattice spacing between the SnO_2_ crystal planes were estimated.

**Figure 1:**
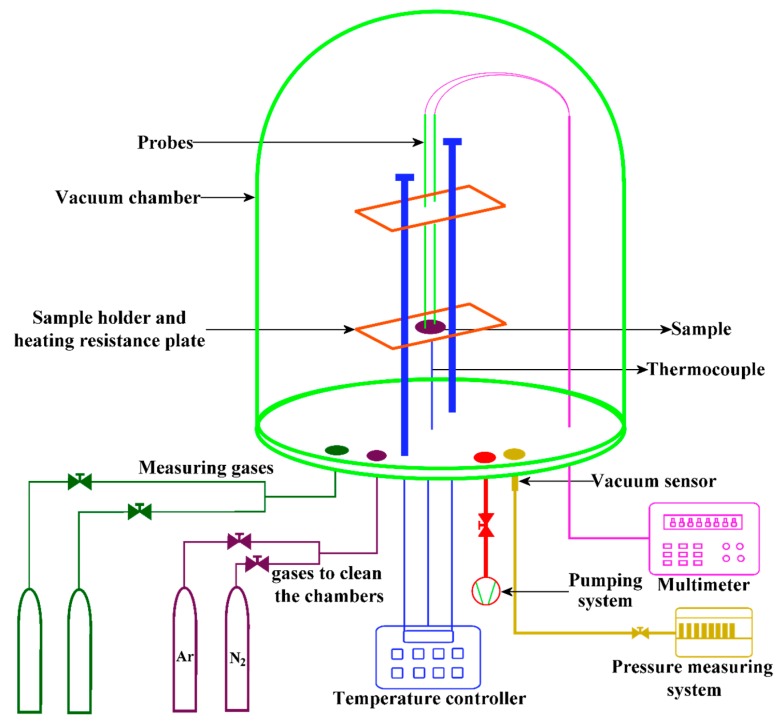
Schematic diagram of the homemade gas sensing system.

For CO sensing measurements, pure silver ohmic contacts were deposited on the pellets surface by the thermal evaporation technique. The experimental setup for measuring the electrical resistance of the pellets is shown in Figure 1. The measurements were made at three different operating temperatures, namely 100, 200, and 300 °C. Lower operation temperatures (<100 °C) do not lead to conductance changes. The conductance changes were registered by using a Keithley 2001 multimeter (Keithley, Mexico City, Mexico). For controlling the partial pressure in the chamber a TM20 detector (Leybold, Mexico City, Mexico) was used. The sensing response of the pellets, S = Rair/Rgas, was obtained by calculating the electrical conductance ratio from the measured resistance in air, Rair, and in the presence of different concentrations of CO, 1, 5, 50, 100, 200 and 300 ppm, Rgas.

## 3. Results

### 3.1. X-ray Diffraction Analysis

**Figure 2:**
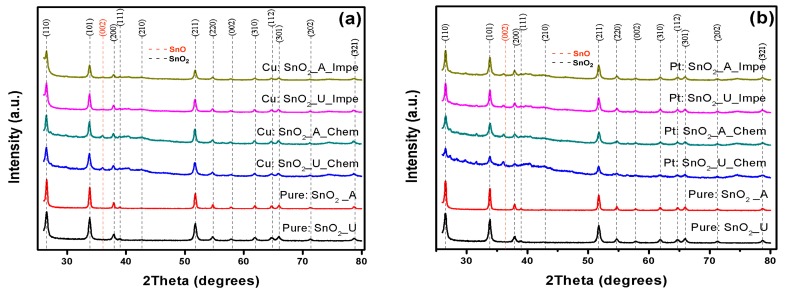

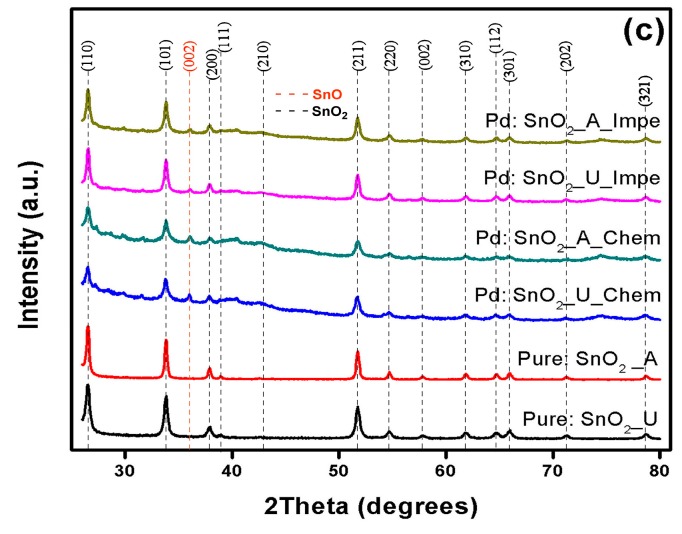
XRD patterns of (**a**) Cu:SnO_2_; (**b**) Pt:SnO_2_; and (**c**) Pd:SnO_2_ powders for different precipitation agents and doping methods.

In this work, doped SnO_2_ nanocrystals were synthesized under various conditions involving different precipitation agents, dopants and doping methods. Figure 2a–c depict the XRD patterns obtained for Cu, Pt and Pd:SnO_2_ powders respectively in comparison with pure SnO_2_ XRD patterns. Irrespective of the precipitation agent and doping method, it is evident from these figures that all the doped-SnO_2_ powders exhibit the tetragonal rutile phase of SnO and SnO_2_, matching the JCPDS cards 06-0395 and 77-0450, respectively [35], whereas, undoped powders demonstrate pure SnO_2_ peaks, and no additional SnO_2_ phases were found. Therefore, the simultaneous presence of SnO and SnO_2_ in all doped powders leads to the conclusion that the dopants reduce the SnO_2_ crystallinity and inhibit the further oxidation of the SnO to SnO_2_, and intergrowth mechanisms may occur at a thermal oxidizing temperature of 800 °C [36].

No additional peaks corresponding to Cu, Pt and Pd are found in the XRD spectra, which can be due to two reasons. First, the doping concentration is 1 wt %, which is very low to detect any changes with the X-ray diffractometer, this affirms a partial incorporation of dopants into the crystal lattice, whereas others can be in form of clusters. Secondly, total incorporation of all dopants into the SnO_2_ crystal lattice in all the doping cases also results an absence of dopant peaks. An upshift and additional peaks were observed in all the doped powders which is corroborated by the decrease in the SnO_2_ crystal quality, and these discrepancies are much higher for Pt and Pd:SnO_2_ as compared to Cu:SnO_2_ powders. The amount of crystal discrepancies is proportional to the ionic radii of the dopants (the ionic radii of Sn^2+^, Cu^2+^, Pt^2+^ and Pd^2+^ are 0.71, 0.73, 0.80 and 0.86 Å, respectively) [26,27].

**Figure 3:**
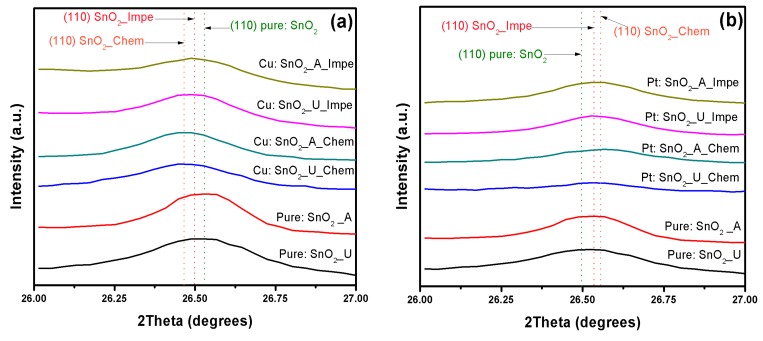

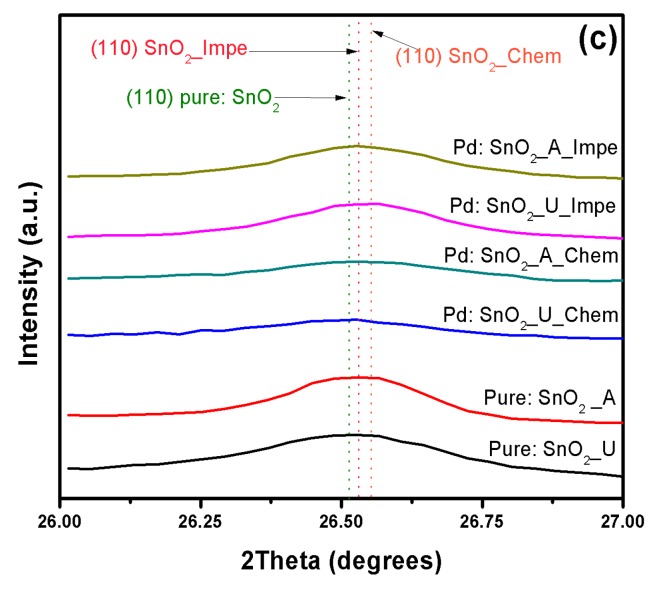
(110) plane shift and peak broadening of (**a**) Cu:SnO_2_; (**b**) Pt:SnO_2_; and (**c**) Pd:SnO_2_ powders for different precipitation agents and doping methods.

Figure 3a–c show the shift in the SnO_2_ preferential orientation plane (110) of the Cu, Pt and Pd:SnO_2_ samples, respectively. Irrespective of the dopant utilized (Cu, Pt or Pd), the chemically doped powders shift slightly to a higher angle compared to impregnated powders. A left shift with peak broadening was observed for Cu:SnO_2_ powders (Figure 3a), while on the contrary a right shift is evidenced for Pt:SnO_2_ and Pd:SnO_2_ (Figure 3b,c). This clearly evidences that the incorporated dopants cuase a strain in the SnO_2_ crystals which results in a planar stress. The shift to a lower angle for Cu:SnO_2_ powders corresponds to a compressive stress and those to a higher angle for the Pt, Pd:SnO_2_ powders represents a tensile stress [26,27].

In order to ascertain the effect of dopants (Cu, Pt and Pd) on the structural characteristics, crystallite size (D) [37], crystal volume (V) [38] and porosity (P) [39] are calculated from Equations (1)–(5) and reported in Table 2.

(1) $$D=\frac{0.89\lambda }{\beta \mathrm{Cos}\theta }$$

(2) $$V=a^{2}c$$

(3) $$P=\left(1-\frac{\rho_{a}}{\rho_{x}}\right)\times 100\%$$

(4) $$\rho_{a}=\frac{m}{v}$$

(5) $$\rho_{x}=\frac{nM}{NV}$$

where *λ* is the wavelength of the incident X-rays (*λ* = 0.15418 nm), *β* is the full width half maximum (FWHM) intensity, *θ* is the Bragg’s diffraction angle in radians, *a* and *c* are lattice parameters, *m* and *v* are mass and volume of the samples, *n* is number of molecules per unit cell, *M* is the molecular weight and *N* is Avogadro’s number.

From Table 2 it can be observed that, for Cu:SnO_2_ powders, the crystallite sized decreased (a left shift, compressed stress; Figure 3) from 26 to 21 nm, whereas for Pt and Pd:SnO_2_ powders crystallite size increased (a right shift, tensile stress; Figure 3) from 26 to ~35 and 42 nm, respectively. These crystallite size changes are in good agreement with the peak shifts observed in Figure 3. The SnO_2_ crystallite size of chemically doped powders decreased more with Cu doping and increased more for Pt and Pd dopants, compared to impregnated powders. The higher ionic radii of Pt and Pd compared to Sn results in a tensile stress and increase in SnO_2_ crystallite size for Pt, Pd:SnO_2_ powders [40]. The presence of Cu inhibited further SnO_2_ growth and decreases the crystallite size, whereas seeing compressed stress for Cu:SnO_2_ powders is puzzling. A linear relation is noticed between the volume of the unit cell and porosity and is apparent because doping with an atom with higher ionic radius increases the volume of the unit cell and subsequently porosity. No significant effects of precipitation agent (urea/ammonia) on structural properties were observed.

### 3.2. Raman Analysis

To confirm the effect of precipitation agent and doping method on the SnO_2_ nanoparticles’ growth, Raman spectroscopy measurements were carried out. It is well known that SnO_2_ has a tetragonal structure with six atoms (two Sn and four O) per unit cell [41]. The 6-unit cell atoms give a total of 18 branches for the vibrational modes in the first Brillouin zone. The mechanical representation of the normal vibration modes, all the vibrational modes of SnO_2_ with corresponding Raman shift peaks were also widely known and reported by various authors [42,43,44]. Table 3 summarizes the different frequencies of the optical modes of SnO_2_, with the corresponding vibrational directions.

**Figure 4:**
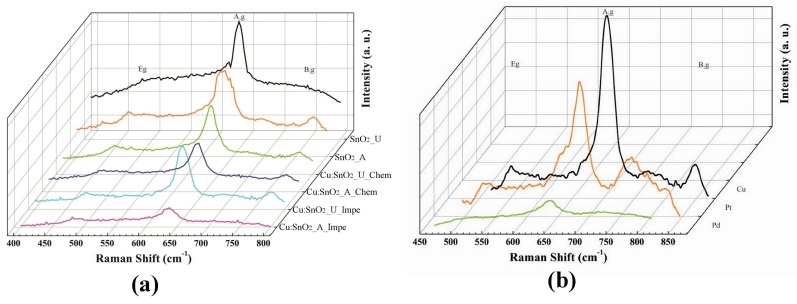
Raman spectra of the (**a**) Cu:SnO_2_ with different doping methods; and (**b**) Cu, Pt and Pd:SnO_2_ using chemical doping with urea as precipitation agent.

The Raman spectra of Cu, Pt and Pd:SnO_2_ synthesized by different doping methods are shown in Figure 4a,b. One IR active and three Raman active modes were observed at 255.2, 482.8, 637.6 and 779.2 cm^−1^, corresponding to the E_u_, E_g_, A_1g_, and B_2g_ vibration modes of SnO_2_, respectively, corresponding in turn to the bulk rutile SnO_2_ [45]. The intensity of the Raman peaks denotes the amount of extraction and compression of Sn-O bonds in the lattice [46]. It is interesting to note that the Cu doping decreases the intensities of Raman peaks (Figure 4a) which is corroborated by the Sn-O disorders arising due to Cu incorporation in the SnO_2_ lattice.

From Figure 4a, it can also be observed that the intensities of the A_1g_ Raman peak were much less for powders synthesized with ammonia as precipitation agent. The rate of decrease in the A_1g_ peak intensity is higher for chemically doped powders than impregnated ones. No peak shifts were observed from the Raman spectra, and the minimum amount of doping percentage for observing a peak shift in Raman spectra was around 3 wt % [43,44,45].

Figure 4b shows a comparison of the Raman spectra of chemically doped Cu, Pt and Pd:SnO_2_ with urea as precipitation agent. Cu:SnO_2_ doped powders present higher intensities than Pt and Pd:SnO_2_ powders which is correlated to the ionic radii of the dopant ions. As Pd has a higher ionic radius compared to Pt and Cu, it causes higher Sn-O disorders in the lattice, which makes the intensities of Pd:SnO_2_ powders diminish. The effect of grain size on the Raman intensities is explained in detail in the upcoming SEM analysis (Section 3.3).

### 3.3. SEM Analysis

**Figure 5:**
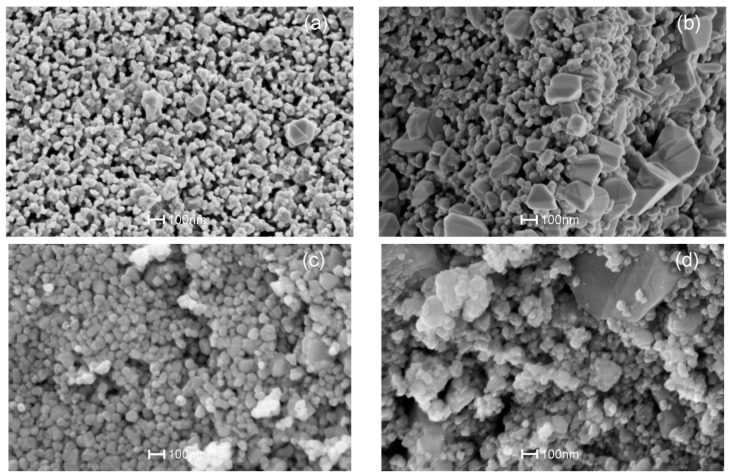
SEM images of Cu:SnO_2_ powders using chemical doping with precipitation agent (**a**) urea, (**b**) ammonia and Cu impregnated SnO_2_ powders with precipitation agent (**c**) urea, (**d**) ammonia.

SEM was employed to further examine and interpret the morphologies and structures of the powders with respect to the precipitation agent and dopant methods. Figure 5a–d show representative SEM images of Cu:SnO_2__U_Chem, Cu:SnO_2__A_Chem, Cu:SnO_2__U_Impe, Cu:SnO_2__A_Impe, respectively. The observed average diameters of the Cu:SnO_2_ nanoparticles are in the 25–35 nm range.

From Figure 5, powders that are chemically doped (Figure 5a,b) shows a contact area between particles, which are the necks between grains. The additives which are added during the synthesis process are incorporated into the lattice of the metal oxide, which further inhibits the grain size [47] and promotes coalescence during the heat treatment. This later coalescence leads to the formation of necks between the grains [48], which in turn influence the bulk conductivity and the thickness of the space charge region. On the other hand, impregnated powders (Figure 5c,d) show only an agglomeration between the particles, the additives which are impregnated on the thermally treated SnO_2_ powders which does not promote the coalescence.

**Figure 6:**
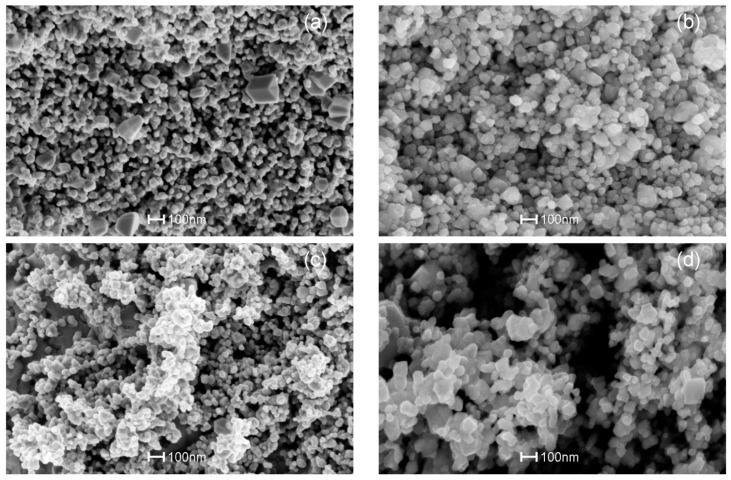
SEM images of (**a**,**b**) Pt:SnO_2_ and (**c**,**d**) Pd:SnO_2_ powders using chemical doping with (**a**,**c**) urea and (**b**,**d**) ammonia as precipitation agents.

From Figure 5b,d, it is also evident that larger crystals around 200–600 nm in size were observed for the powders with ammonia as precipitation agent in addition to the SnO_2_ nanoparticles. The formation of larger crystals is due to the rapid precipitation at room temperature. In the case of powders precipitated using urea (Figure 4a,c), the elevated temperature of around 90 °C utilized for decomposing the urea also resulted in homogenous precipitation [49]. This in turn resulted in a relatively uniform particle size of around 20 nm and similar behavior was noticed for the Pt and Pd:SnO_2_ powders (Figure 6). Figure 6a–d show representative SEM images of Pt:SnO_2_ and Pd:SnO_2_ powders obtained by chemical doping and impregnation, respectively. 

Like the Cu:SnO_2_ powders, Pt and Pd:SnO_2_ powders also shows necks between the grains for the chemically doped ones (Figure 6a,c). The average diameters of the observed Pt and Pd:SnO_2_ nano-particles are around 45 and 60 nm, respectively. The structures shown in Figure 5 correspond to Cu:SnO_2_, whereas the structures shown in Figure 6 corresponds to Pt and Pd:SnO_2_ powders, whose particle and grain sizes are relatively larger than the former ones. This increase is corroborated to the collection of grain boundaries by the additives due to the interfacial tension [47]. It is well known that the lower interfacial tension involves less energy in formation of a surface, which subsequently gives lower size grains [47,48,49]. Pt and Pd need a higher interfacial tension due to their higher ionic charge compared to copper [48], which results in more disorder in Pt- and Pd-doped SnO_2_ structures, as evidenced by the Raman analysis (Figure 4b). From the Raman analysis (Figure 4b), we can observe that the Cu:SnO_2_ powders show higher Raman intensities than Pt and Pd:SnO_2_ powders, corresponding to a lower Sn-O disorder. From Figure 5 and Figure 6, we can conclude that the chemically doped powders are favorable to promote gas sensing because the conductivity increases due to the formation of necks between the grains [50].

**Figure 7:**
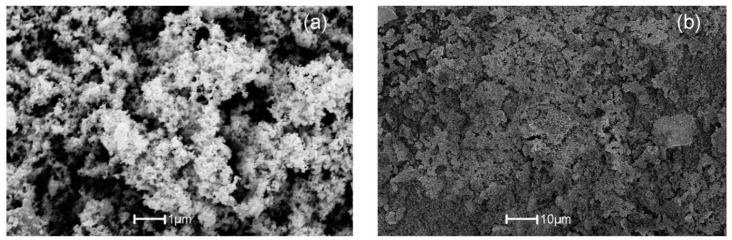
SEM images of Cu:SnO_2_ using chemical doping with urea as precipitation agent at (**a**) 1 µm; and (**b**) 10 µm scales.

Porosity is another important factor that affects the sensing response [51]. It has already been reported [52,53] that the SnO_2_ sensing response increases with respect to the porosity with different additives. An increment in the surface porosity will increase the active surface area which allows the sensing gas to diffuse into the pellet pores [54]. To observe the pellet surface porosity, chemically doped Cu:SnO_2_ powders were observed by SEM at a lower magnification and the results are shown in Figure 7. The porous surface has a mesh-like morphology due to the smaller particle size and formation of necks between the particles.

### 3.4. HRTEM Analysis

**Figure 8:**
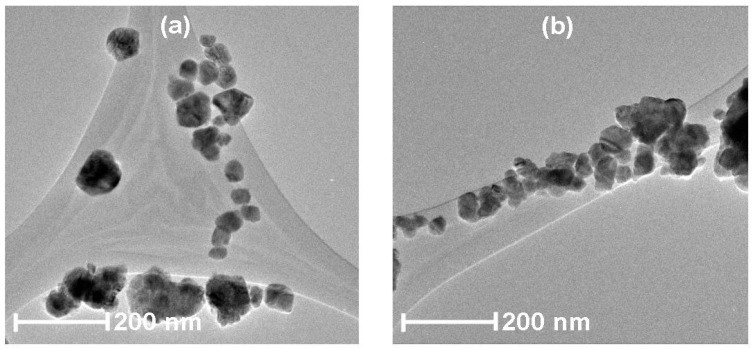

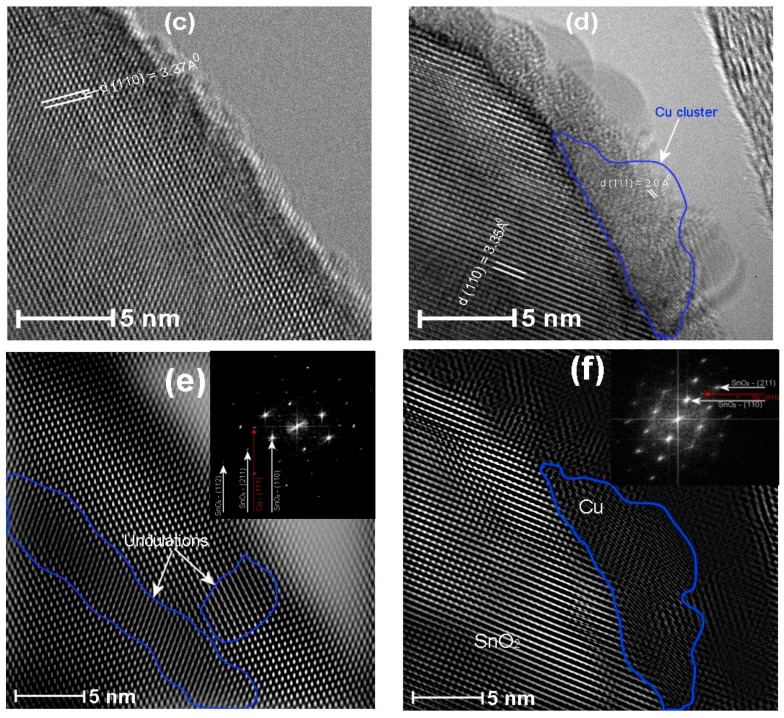
HRTEM images of Cu:SnO_2_ prepared by (**a**) chemical doping with urea as precipitation agent; (**b**) impregnation with ammonia as precipitation agent; a higher magnification of surface of SnO_2_ crystal surface of (**a**,**b**) is shown in (**c**,**d**). (**e**) Reconstructed HRTEM image after masking of (**c**); inset: the corresponding SAED pattern, and (**f**) reconstructed HRTEM image after masking of (**d**); inset: the corresponding SAED pattern.

To obtain more-detailed structural information the Cu-, Pt- and Pd-doped SnO_2_ particles were analyzed by HRTEM. Figure 8 shows the Cu:SnO_2_ particles prepared by chemical doping (a) and impregnation (b). The powders are composed of agglomerated nanometric crystals around 25–30 nm in size, which is in good agreement with the XRD results.

Magnified views of Figure 8a,b are shown in Figure 8c,d and their corresponding reconstructed images are shown in Figure 8e,f, respectively. The insets in Figure 8e,f are the corresponding selected-area electron diffraction (SAED) patterns. The differences in the effects of the doping method (chemically doped/impregnated) are remarkable. In the case of the chemically doped sample, all the atomic planes are well defined and correspond to SnO_2_ crystals (Figure 8c) and its surface is free from any dopants, unlike for impregnated powders (Figure 8d). The *d*-spacings measured from SAED are in good agreement with those of the (110) plane of cassiterite SnO_2_ (JCPDS Card 77-0450 card) [35], corresponding to the tetragonal crystal structure (space group = *P42/mnm*).

Cu doping localized on the surface of the SnO_2_ particle was observed for the impregnated powders (Figure 8d,f), marked in the blue region. Also, no defects were founded in the SnO_2_ crystal. The inter-plane distance measured for the copper particle corresponds to the (111) plane of cubic Cu (JCPDS Card 00-004-0836) [55], which confirms that the cluster formation of the dopants takes place on the surface of the SnO_2_, which assures the possibility of a spillover mechanism in the gas sensing properties.

Contrarily, for chemically doped powders, no clusters of dopants were observed on the surface but the surface undulations produced by doping were observed and are marked in Figure 8e, highlighted in the blue regions. Therefore, when using the chemical doping method, the dopants are incorporated into the SnO_2_ lattice. Moreover, the (110) inter-plane distances were estimated to be 3.35, 3.37 and 3.36 Å for undoped, chemically doped and impregnated powders, respectively. This increase in the lattice parameter suggests an increase of the unit cell volume for the doped powders, which is a cogent evidence for Cu insertion in the host (SnO_2_) matrix. The estimated lattice spacing values from the HRTEM are consistent with the calculated XRD data. A comparison of the calculated d-spacing values for all the dopants and doping methods is tabulated in Table 4. The change in the lattice spacing is slightly higher in chemically doped powders, which is consistent with the higher left shift in the (110) diffraction peak (Figure 3).

**Figure 9:**
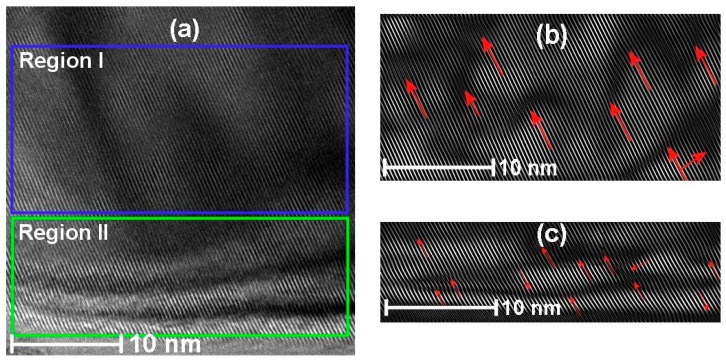
HRTEM images of Cu:SnO_2_ using chemical doping with urea as precipitation agent (**a**) surface of the SnO_2_ crystal with stacking faults; (**b**) reconstructed image of Region I; and (**c**) reconstructed image of Region II.

Figure 9a shows the surface of the Cu:SnO_2_ crystals divided into regions I and II, with their corresponding reconstructed (Figure 9b,c) patterns. Figure 9b,c evidence the stacking faults (marked with red arrows) in the SnO_2_ lattice, which are attributed to the Cu doping. 

In “Region I” from Figure 9 these faults are consistently observed on all surfaces of chemically doped SnO_2_ powders. The numbers of defects produced were more in the case of Pt and Pd than with Cu, which is due to the higher ionic radii of Pt and Pd.

**Figure 10:**
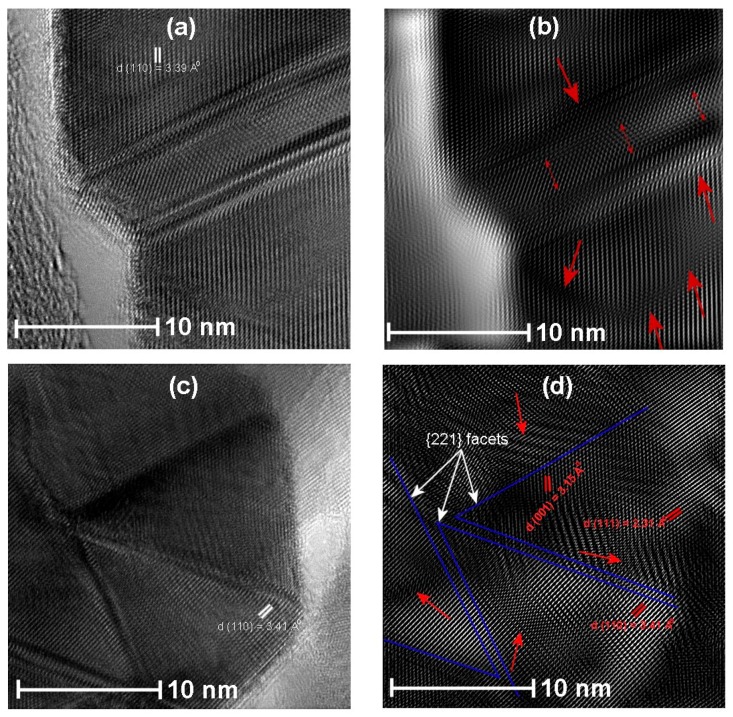
HRTEM images of (**a**) Pt:SnO_2_ prepared by chemical doping with urea as precipitation agent, (**b**) Reconstructed HRTEM image after masking of (**a**), (**c**) Pd:SnO_2_ prepared by chemical doping with urea as precipitation agent and (**d**) Reconstructed HRTEM image after masking of (**b**).

Figure 10a,c show the defects produced for the SnO_2_ powders chemically doped with Pt and Pd, respectively. FFT reconstructed images in Figure 10a,c are shown in Figure 10b,d, respectively. For Pt doped SnO_2_ powders the stacking faults obtained are in the (110) plane surface, which are marked with red arrows, whereas, in case of Pd:SnO_2_ powders, as shown in Figure 10d, the stacking faults or defects are obtained on the {221} facets of SnO_2_ projected from (110) plane. The {221} place is a typical facet of SnO_2_ and can be described as a combination of (001) and (110) steps [56]. 

According to Han et al. [56], {221} facets are very favorable for gas sensing properties. Further detailed chemical analysis of the Pd:SnO_2_ crystals is required to explain the reasons for the formation of {221} facets. The calculated (110) inter-plane distance was 3.39 and 3.41 Å for chemically doped Pt and Pd:SnO_2_ powders, respectively. The presence of dislocations and stacking faults observed in the HRTEM analysis agrees with the Raman bands of Cu-, Pt- and Pd-doped SnO_2_ powders, which show an asymmetric broadening A_1g_ Raman peak (cf. Figure 4b). The internal strain produced due to the doping leads to a downshift and broadening of the A_1g_ Raman peak [57]. Additionally, due to the stacking faults, the crystals obtained have Sn-enriched stoichiometry in addition to Sn-interstitial spaces and oxygen vacancies [58], therefore, the increment in the [Sn]/[O] ratio at the stacking fault causes electronic effects, which increases bulk conductivity and subsequently a CO sensing response.

### 3.5. CO Sensing Properties

**Figure 11:**
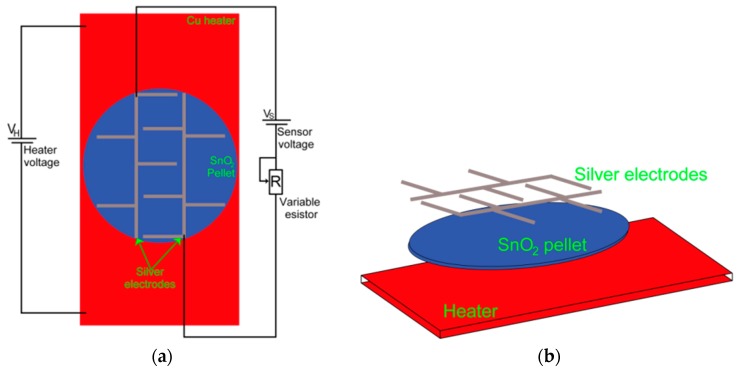
(**a**) Top view and (**b**) cross sectional view of SnO_2_ sensor.

**Figure 12:**
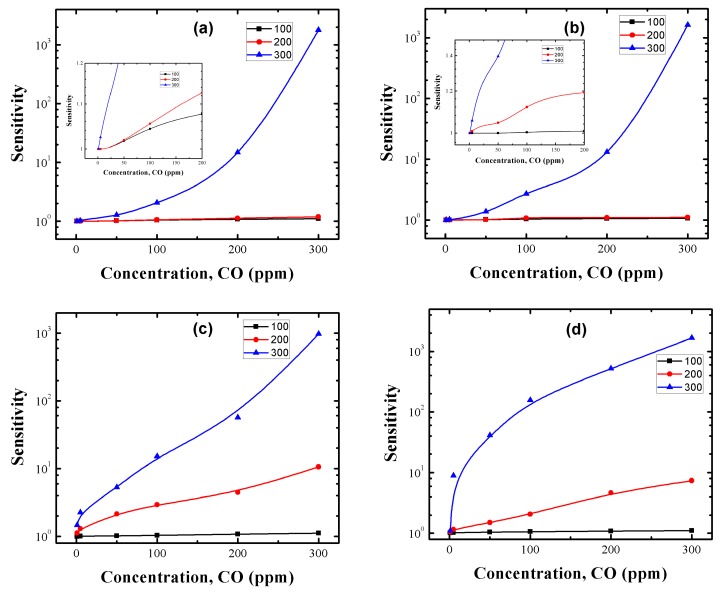
Sensing response of Cu:SnO_2_ powders using chemical doping with precipitation agent (**a**) urea; (**b**) ammonia and Cu impregnated SnO_2_ powders with precipitation agent (**c**) urea; (**d**) ammonia.

CO gas sensing measurements were performed for the manufactured Cu-, Pt- and Pd-doped SnO_2_ pellets to taste their potential applications in chemical sensors. Top and cross sectional views of the gas sensor employed in this work are shown in Figure 11a,b, respectively. To study the effect of precipitation agent, dopants and doping method on the measuring temperature and CO gas concentration of our SnO_2_ samples, the sensor response was tested as a function of CO gas in the range of 1–300 ppm at temperatures of 100, 200 and 300 °C. Figure 12a–d correspond to the CO sensing properties of Cu:SnO_2__U_Chem, Cu:SnO_2__A_Chem, Cu:SnO_2__U_Impe and Cu:SnO_2__A_Impe, respectively. Table 5 summarizes the highest sensing responses obtained at 300 °C for 300 ppm of CO gas concentration, for the different dopants used, synthesis routes and doping methods. The undoped sensing responses are reported from our previous work [34].

Irrespective of the dopant used, doping method and synthesis route employed, the sensing responses of doped SnO_2_ pellets are higher than for undoped SnO_2_ (Table 5). From Figure 12a–d and Table 5, it can be observed that irrespective of the synthesis route and doping method employed, all the pellets’ sensing responses increase with an increment in the gas concentration and measuring temperature.

Cu:SnO_2__U_Chem, Cu:SnO_2__A_Chem, Cu:SnO_2__U_Impe and Cu:SnO_2__A_Impe also exhibits a rapid response increase to reach a maximum value of 1782, 1625, 975 and 1666, respectively, at the measuring temperature of 300 °C. Chemically doped powders show a slight increase in sensing response when measured at 100 and 200 °C (cf. insets of Figure 12a,b), whereas the impregnated powders show an effective sensing response at the measuring temperature of 200 and 300 °C (Figure 12c,d). Finally, only at higher gas concentrations like 300 ppm, do pellets manufactured with urea as precipitation agent show higher sensing responses than powders utilizing ammonia as precipitation agent (Table 5). Additionally, irrespective of the precipitation agent utilized, the sensing responses obtained are higher for impregnated Cu:SnO_2_ powders than chemically doped powders at low concentrations (1–50 ppm) and for lower temperatures (200 °C) and the response shows an increasing tendency with an increase in CO concentration, for all the measuring temperatures (Figure 12a,b insets).

**Figure 13:**
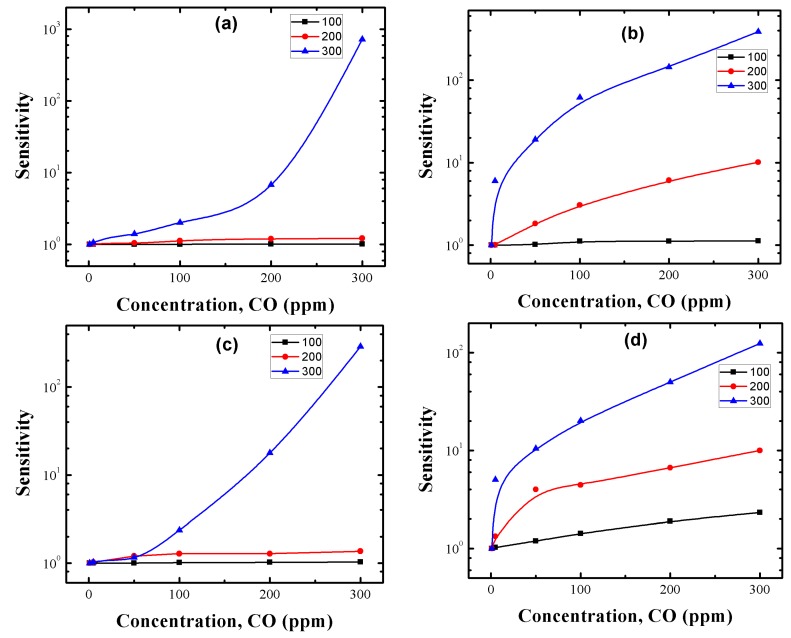
Sensing response of (**a**,**b**) Pt:SnO_2_ and (**c**,**d**) Pd:SnO_2_ powders using (**a**,**c**) chemical doping and (**b**,**d**) impregnation method with urea as precipitation agent.

To study the effect of the dopant type on the CO sensing responses, the sensor responses of the Pt- and Pd-doped SnO_2_ pellets were tested taking into consideration the different doping methods, and results are reported in Figure 13. We can observe that Pt- and Pd-doped pellets are in the sensing response range of the Cu-doped pellets. The sensing responses increase with respect to temperature and gas concentration for both Pt- and Pd-doped samples. Additionally, impregnated powders showed relatively higher sensing responses at lower concentrations (0–50 ppm) and lower temperatures (200 °C). Finally, the sensing responses of Pt- and Pd-doped SnO_2_ samples are smaller compared to Cu-doped samples. Chemically doped powders show higher sensing responses than the impregnated ones for all Cu, Pt and Pd:SnO_2_ powders (Table 5) at higher CO concentrations and this trend is reversed for lower CO gas concentrations.Table 5 summarizes the sensing responses of the Cu, Pt and Pd:SnO_2_ pellets prepared by different doping methods and precipitation agents, measured at 300 °C for 300 ppm of CO. We can observe that Cu shows higher sensing responses that Pt and Pd in each case. The sensing response increases with respect to the measuring temperature and gas concentration. The detailed explanation of the above obtained results and their corresponding reasons will be discussed in the upcoming.

## 4. Discussion 

### Gas-Sensing Mechanism

**Figure 14:**
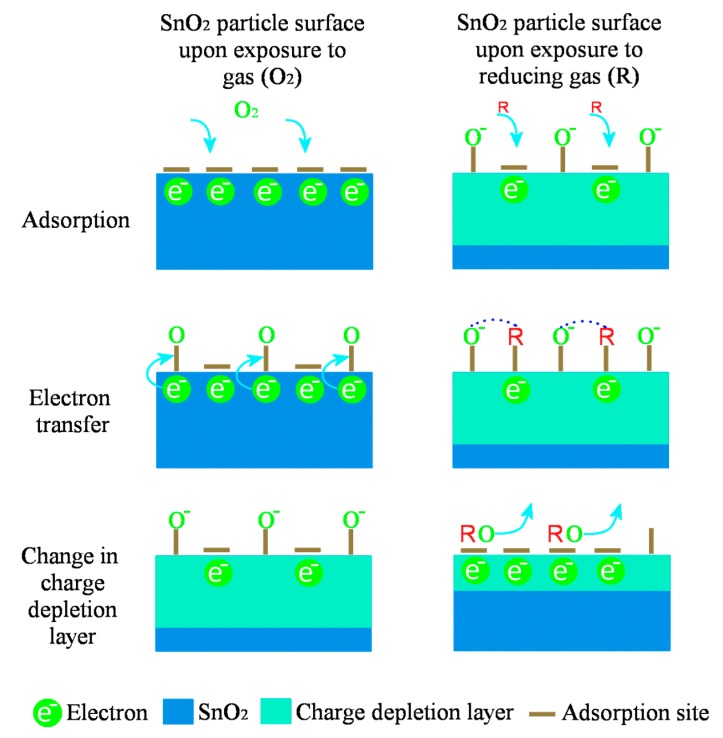
Schematic indicating the sensing mechanism on the SnO_2_ surface. Left column indicates reaction with oxygen, whereas right column shows the interaction with reducing gas like CO.

A description of the oxygen adsorption on a metal oxide with a change in surface conductivity with respect to CO is presented in Figure 14. As the temperature increases, atmospheric oxygen adsorbs on the SnO_2_ surface due to its oxygen vacancies which results in the formation of a depletion region between adsorbed oxygen and SnO_2_ surface (left column of Figure 14). Later, as the reducing gas interacts with the adsorbed oxygen and increase the conductivity which is measured as a sensor signal (right column of Figure 14).

**Figure 15:**
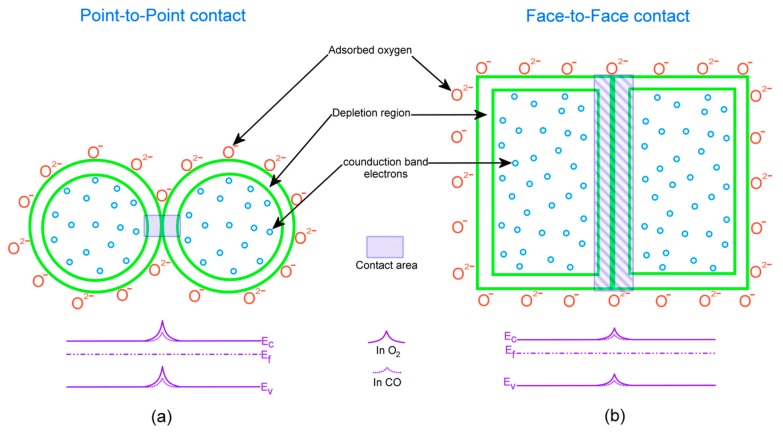
Illustration of the contact area for (**a**) spherical grains; and (**b**) tetragonal grains.

A rise in the CO gas concentration leads to an increment in the number of reactions with the pellet surface, which in turn increases the magnitudes of conductivity and subsequently the sensing response. Also, the oxygen species adsorbed at temperatures above 300 °C is atomic (O^−^) which is more reactive, whereas the adsorbed oxygen at 100 and 200 °C temperatures is in molecular form (O_2_, O^2−^) [59]. Therefore, the sensing response increases accordingly to the gas concentration and temperature which is in consistent with our results reported in Figure 12 and Figure 13. From the obtained SEM and HRTEM results, it is evident that the structures obtained are non-spherical and porous, which provides an additional active sites to adsorb more oxygen species which increased the sensing response [60]. Figure 15 indicates the difference between the contact area for spherical and non-spherical contacts. For illustrative purposes, considering that the non-spherical structures formed a tetragon (which is a typical phase of SnO_2_), this results in a different contacting form with different contact area.

As shown in Figure 15a, for spherical structures point-to-point contacting consists of relatively less contact area compared to the tetragonal structures with the face-to-face contact as shown in Figure 15b. When different tetragonal particles are exposed to air, oxygen adsorption between the face-to-face contact results in a less resistive depletion region between the adjacent particles compared to the point-to-point contact. The face-to-face contacting form of tetragonal particles enhances the surface-depletion control rather than grain boundary barrier control, which results in a higher sensing response.

Compared to the state of art of SnO_2_-based sensors [20,25,29,31,51,58,60], the CO sensing response obtained in this work is much higher. A summary of the sensing responses is presented in Table 6. The main aim of comparing sensing responses of different gases and temperatures is to convey the idea that the sensing responses obtained in this work are much higher due to the presence of adequate material resulting from the usage of pellet sensors. The utilization of pellets increased the sensing response values which in the future can served as a reference to compare the responses of thin and thick films sensor. The maximum sensing response obtained for CO was around 100 and for H_2_S it was around 357. The minimum operation reported in Table 6 was around 100 °C. In our work, we have obtained an CO sensing response increased by one order of magnitude (1782) for Cu:SnO_2_ powders for very low concentrations of CO.

For chemically doped powders, the incorporation of additives in the SnO_2_ lattice increases the charge carrier concentration, which subsequently increases the bulk conductivity and decreases the thickness of the space charge region. This effect is clearly observed from our SEM results (Figure 5). During the thermal treatment, the presence of dopants gives rise to two important factors, coalescence of the SnO_2_ and modification of the crystal structure [17,50]. 

Due to coalescence, we can observe an increase of the contact area between crystallites and the formation of necks between grains. This will lead to formation of a sensor matrix network [16,69], which is obtained for chemically doped powders. Modification of crystallographic structure leads to an increase in crystal defect concentration and a change in crystallographic faceting, and these changes will enhance the conductivity of the SnO_2_ matrix [70,71]. It is evident from the HRTEM results (Figure 8, Figure 9 and Figure 10) that stacking faults for Cu and Pt:SnO_2_ powders and {221} facets for Pd:SnO_2_ powders are observed. It was recently shown by Han et al. [56], that {221} facets show higher sensing responses compared to others. Therefore, the formation of necks and the structural modifications produced by chemical doping enhanced the sensing response of the doped SnO_2_ pellets. Crystallite size obtained for powders with urea as precipitation agent were relatively uniform and homogenously distributed, which increased the surface to volume ratio and further the sensing response [72].

**Figure 16:**
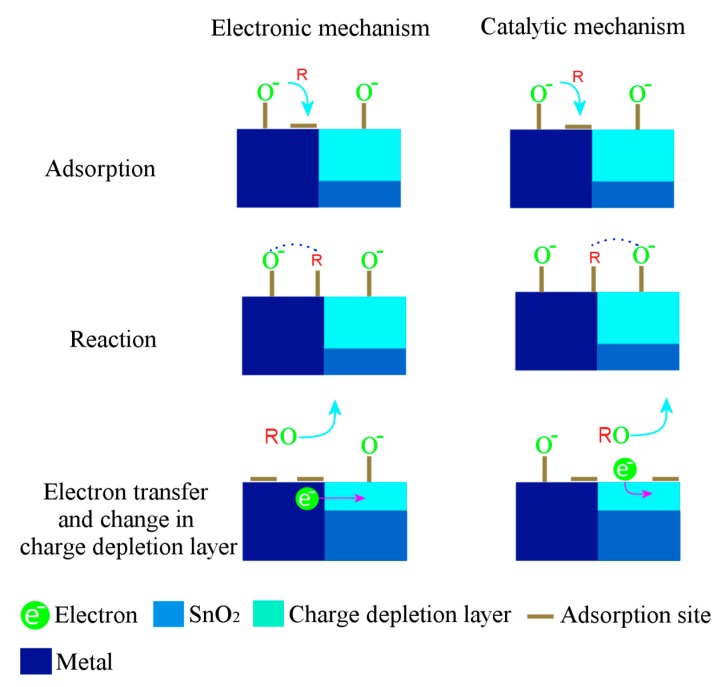
Schematic diagram indicating the general steps involved in the electronic (left column) and catalytic (right column) mechanisms active in SnO_2_ sensors with metal additives. *R* represents a reducing gas.

The sensing response of the impregnated powders is due to the formation of clusters on the surface of the SnO_2_ crystals (Figure 8d,f). Therefore, two types of sensitization mechanisms, chemical and electronic, are considered for explaining the effect of clusters on the sensing response. Detailed explanations of both mechanisms have been discussed by many authors previously [73,74,75], Figure 16 shows both mechanisms in detail. In the electronic mechanism, there is only electronic interaction between the semiconductor and cluster and these interactions change depending on the form of the cluster. It has been evidenced that metal clusters like Cu, Pt and Pd in oxide from can only interact electronically with the SnO_2_ and these interactions vanish if the clusters are metallic [60]. Since no oxides were observed from our HRTEM results, the electronic mechanism can be ruled out. To the best of our knowledge, until now the previous reports [46,75] suggest that the spillover effect (chemical mechanism) lowers the measuring temperature, which could be the possible reason for the increased sensing response for our impregnated powders at 200 °C (Figure 12 and Figure 13).

Another parameter observed from Figure 12 and Figure 13 is that Cu, Pt and Pd:SnO_2_ powders present higher sensing responses for chemically doped samples only at higher CO concentrations (100–300 ppm). Impregnated powders show higher sensing responses for low CO concentrations (0–50 ppm). This shows that the impregnated powders show an exponential increase and a saturation tendency with increased gas concentration, whereas for chemically doped powders no saturation is shown until 300 ppm of CO. Therefore, it can be concluded that the clusters formed in the impregnation method favor the detection of very small concentrations of CO and saturates with lower sensing responses, whereas chemically doped powders require a minimum of 50 ppm of CO for detection, but the response is relatively higher at higher gas concentrations.

**Figure 17:**
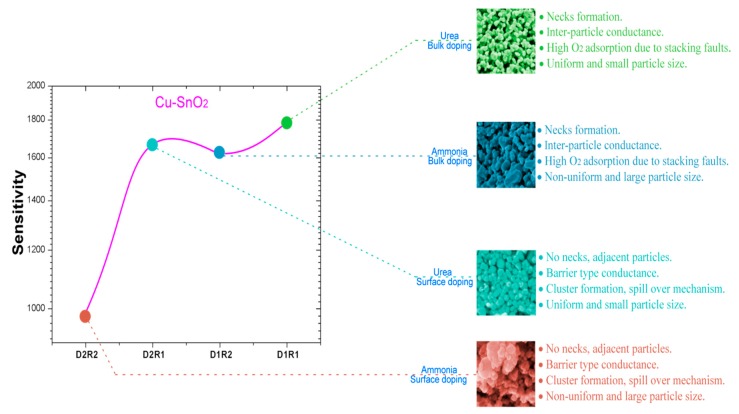
Graphical comparison of synthesis methods of Cu:SnO_2_ pellets with corresponding intellects for achieving the high sensing responses for higher CO concentrations (0–300 ppm).

Another crucial factor that influences the sensing response is the doping concentration of the additives. In this work, we have utilized 1 wt % of concentration for all samples. It has been established that the response of SnO_2-_based sensors lies in the range of 0.1–0.6% [41] for noble metals and for the transition metals it is in the range of 1–4% [48]. However, as we observe from the results obtained in this work, we can conclude that the sensing response depends on many factors like the measuring temperature, microstructure, crystallite size and shape, dopants and on the interaction between the sensing matrix and gas molecules. Further work is necessary to find the exact role of transition and noble metals as dopants on the CO sensing response. A comparison of doping methods and precipitation agents utilized in this work, with their corresponding advantages and sensing responses for only higher CO concentrations of Cu:SnO_2_ pellets was represented graphically in Figure 17. Lower gas concentrations shown are not well understood due to a very different behavior with respect to the doping methods utilized. A more detailed study will be performed in our future work to determine the effect of gas concentration on the doping method. From Table 5, irrespective of the doping method and synthesis route, Cu as dopant showed higher sensing responses than Pt and Pd, which is an interesting and novel result. Therefore, doping of Cu, a transition metal, in SnO_2_ powders at 1 wt % doping produced better sensing response magnitudes in this work than noble metal dopants like Pt and Pd. From this result, it can be concluded that the fabrication of Cu:SnO_2_ sensors will be a relatively cost effective design method, while featuring a higher CO sensing response magnitude.

## 5. Conclusions

In this work, Cu-, Pt- and Pd-doped SnO_2_ powders were successfully synthesized utilizing chemical doping and impregnation methods. Cu-, Pt- and Pd-doped SnO_2_ powders crystallite size and porosity was increased, respectively, compared to the undoped powders, due to the increase in the ionic radii of the dopants. A shift in the (110) SnO_2_ plane confirms the incorporation of dopants in the SnO_2_ crystal lattice. The intensity of the Raman peaks decreased in the order of undoped, Cu, Pt and Pd:SnO_2_. This decrease corresponds to the amount of extraction and compression of Sn-O bonds inside the lattice. Particles sizes obtained by SEM were around 25–35 nm for all the Cu-, Pt- and Pd- doped powders. HRTEM analysis of dopant positions confirms the stacking faults and cluster formation for the powders obtained by chemical doping and impregnation, respectively. Sensing responses of Cu:SnO_2_powders are much higher compared to Pt and Pd:SnO_2_ powders. A drastic decrease in the crystal quality and a substantial number of defects for Pt and Pd:SnO_2_ powders reduced their sensing responses in comparison with Cu:SnO_2_ powders. Irrespective of the doping method, pellets manufactured from the powders synthesized utilizing urea showed high sensing responses than those synthesized using ammonia. Additionally, chemically doped sample sensing responses are higher than impregnated ones. The face-to-face contact form of tetragonal particles enhances the surface-depletion control rather than grain boundary barrier control, which results in a high sensing response in Cu:SnO_2_ powders. The maximum sensing responses obtained was for Cu-doped pellets and was around 1782 for powders incorporated by the chemical method with urea as precipitation agent.

## Figures and Tables

**Table 1:** List of samples with their corresponding doping methods and precipitation agents employed.

Name of the Sample	Doping Method	Precipitation Agent	Dopant
Cu:SnO_2__U_Chem	Chemical doping	Urea	Cu
Cu:SnO_2__A_Chem	Chemical doping	Ammonia	
Cu:SnO_2__U_Impe	Impregnation	Urea	
Cu:SnO_2__A_Impe	Impregnation	Ammonia	
Pt:SnO_2__U_Chem	Chemical doping	Urea	Pt
Pt:SnO_2__A_Chem	Chemical doping	Ammonia	
Pt:SnO_2__U_Impe	Impregnation	Urea	
Pt:SnO_2__A_Impe	Impregnation	Ammonia	
Pd:SnO_2__U_Chem	Chemical doping	Urea	Pd
Pd:SnO_2__A_Chem	Chemical doping	Ammonia	
Pd:SnO_2__U_Impe	Impregnation	Urea	
Pd:SnO_2__A_Impe	Impregnation	Ammonia	

**Table 2:** **C**rystallite size (D); Volume of the crystal (V); and Porosity (P) of the Cu, Pt and Pd-SnO_2_ nano crystals.

Sample Name	D (nm)	V (10^−24^ cm^3^)	P (%)
SnO_2__U	26.3	71.4	32.3
SnO_2__A	30.0	71.6	32.4
Cu:SnO_2__U_Chem	21.2	71.7	58.4
Cu:SnO_2__A_Chem	21.0	71.7	58.5
Cu:SnO_2__U_Impe	22.2	71.7	58.5
Cu:SnO_2__A_Impe	22.0	71.8	58.7
Pt:SnO_2__U_Chem	35.0	71.8	64.6
Pt:SnO_2__A_Chem	35.0	71.8	64.7
Pt:SnO_2__U_Impe	33.0	71.8	64.8
Pt:SnO_2__A_Impe	33.0	71.9	64.8
Pd:SnO_2__U_Chem	42.0	72.0	73.0
Pd:SnO_2__A_Chem	42.0	72.0	73.0
Pd:SnO_2__U_Impe	40.0	72.0	73.0
Pd:SnO_2__A_Impe	40.0	72.2	73.2

**Table 3:** Raman, IR and other vibrational modes of SnO_2_.

Modes	Notation	Direction of Vibration with Respect to c-Axis	Raman Shift (cm^−1^)
**Raman active**	A_1g_	Perpendicular	638
	B_1g_	Perpendicular	100
	B_2g_	Perpendicular	782
	E_g_	Parallel	476
**IR active**	A_2u_	Parallel	705
	E_u_	Perpendicular	244
**Silent**	A_2g_	Perpendicular	398
	B_1u_	Parallel	140

**Table 4:** A comparison of estimated d-spacing values for all the dopants and doping methods.

Dopant	SnO_2_–d (110) in Å					
	Urea	Ammonia	Chemically Doped		Impregnated Powders	
			Urea	Ammonia	Urea	Ammonia
**Undoped**	3.35853	3.35492	-	-	-	-
**Cu**	-	-	3.3732	3.36927	3.3682	3.3927
**Pt**	-	-	3.39422	3.38839	3.3801	3.35427
**Pd**	-	-	3.41855	3.40501	3.40697	3.35706

**Table 5:** Sensing responses at 300 °C for 300 ppm of CO of the Cu-, Pt- and Pd-doped SnO_2_ pellets prepared by different methods.

	Undoped	Cu	Pt	Pd
**U_Chem**	12	-	-	-
**A_Chem**	10	-	-	-
**U_Chem**		1782.609	1200	502.5
**A_Chem**		1625	721.519	287.6405
**U_Impe**		1666.667	428.7234	245.4546
**A_Impe**		975.7412	387.3333	224.1485

**Table 6:** Representative synthesis methods, material characteristics, and corresponding sensing responses for SnO_2_ based sensors compared to present work.

SnO_2_ Synthesis Method	SnO_2_ Precursor	Crystallite Size (nm)	Sensing Response (Ra/Rg at Specified Operating Temperature and Mole Fraction of Target Gas)	Reference
Vapor−liquid−solid	Sn Powder	~75–90	~ 80 at 300 °C and at 10 ppm of CO	[61]
Hydrothermal process	SnCl_2_·2H_2_O	~10	~ 78 at 220 °C and at 100 ppm of acetone	[62]
Hydrolysis and Precipitation	SnCl_4_·5H_2_O	~15	~80 at 350 °C and at 600 ppm of CO	[63]
Chemical spray pyrolysis	SnCl_4_·5H_2_O	~10	~132 at 225 °C and at 400 ppm of NO_2_	[64]
Precipitation	SnCl_4_·5H_2_O	~14	~100 at 350 °C and at 600 ppm of CO	[65]
One-step Solvothermal route	SnCl_4_·5H_2_O	~10	~22.69 at 260 °C and at 50 ppm of ethanol gas	[66]
Sol-Gel	SnCl_4_·5H_2_O	~8–20	~1.95 at 100 °C and at 5 ppm of CO	[67]
Hydrothermal synthesis	SnCl_4_·5H_2_O	~3.4 ± 0.8 nm	~357 at 100 °C and at 5 ppm of H_2_S	[68]
Present work	SnCl_4_·5H_2_O	~35	~1782 at 300 °C and at 300ppm	--
